# The General Mechanism of Status: The Impact of Perceptual and Knowledge-Based Social Status on Selective Attention

**DOI:** 10.3390/bs16050807

**Published:** 2026-05-18

**Authors:** Yunfei Gao, Sicen Zhou, Jingjing Zhao, Yonghui Wang

**Affiliations:** 1 School of Psychology, Shaanxi Normal University, Xi’an 710062, China; gyyunfei@126.com (Y.G.); zhousicen97@163.com (S.Z.); 2 Shaanxi Provincial Key Laboratory of Behavior and Cognitive Neuroscience, Xi’an 710062, China

**Keywords:** social status, object-based attention, space-based attention, SOA

## Abstract

Social status, a fundamental dimension of human social life, shapes access to resources and opportunities and influences how individuals perceive and interact with others. Perceivers can infer others’ social status from both perceptual cues (e.g., clothing) and knowledge-based cues (e.g., biographical information), yet it remains unclear whether and how these distinct types of status cues influence early-stage attentional selection. To address this question, the present study employed a modified two-rectangle paradigm to examine the effects of social status on space- and object-based attention. Social status was manipulated via perceptual cues (Experiment 1: upper-body clothing) and knowledge-based cues (Experiment 2: fictive biographies), and three stimulus onset asynchronies (SOAs: 100, 300, and 500 ms) were included to capture the temporal dynamics. The results showed that both perceptual and knowledge-based status selectively modulated object-based attention at the 300 ms SOA, with comparable effect magnitudes, whereas space-based attention remained unaffected. These findings suggest that social status operates as a higher-order modulatory factor that selectively influences object-based attentional selection within a temporally constrained window. Moreover, the comparable effects of perceptual and knowledge-based cues support the notion that different types of status cues are integrated into a general representational format that guides attentional priority.

## 1. Introduction

An employee can recognize their boss’s face in just 2 s but takes 19 s to recognize their own. This real incident occurred during a 2013 awards ceremony. Coincidence? Unlikely. It reflects a common phenomenon whereby social status influences attentional allocation to faces (Freeman et al., 2013). Social status, which is mainly defined by factors such as occupation, income, and education level (Anderson et al., 2015; Côté, 2011; Kraus & Keltner, 2009; Kraus et al., 2017; Rahal et al., 2021), strongly influences what individuals attend to. Individuals tend to rely on two types of cues to infer others’ status (Anderson et al., 2015; Kraus et al., 2017). Perceptual status cues refer to visually observable information, such as clothing and possessions. In contrast, knowledge-based status cues involve verbal or semantic information, such as biographical descriptions. Using these cues, individuals can quickly and accurately identify others’ social status, thereby supporting effective social interactions (Bjornsdottir & Rule, 2017; Mattan & Kubota, 2020; Oh et al., 2020).

Evolutionary perspectives posit that adaptive psychological mechanisms affect cognition across all levels of processing, from relatively automatic lower-level processes such as attention and memory to more complex processes including decision-making and moral judgment (Cosmides & Tooby, 1994; Kenrick et al., 2002; Kurzban & Leary, 2001). However, previous research on social status has predominantly focused on higher-level cognitive processes, particularly decision-making (Griskevicius et al., 2010; Han et al., 2010; Lee et al., 2018; Mattan et al., 2017; Sheehy-Skeffington, 2020) and social evaluation (Bjornsdottir & Beacon, 2024; Bjornsdottir & Rule, 2017; Durante et al., 2017; Fiske et al., 2002; Freeman et al., 2011; Jung et al., 2023; Kuppens et al., 2018; Mattan et al., 2019; Oh et al., 2020). Few studies have examined the impact of social status on early attentional allocation. The existing studies have manipulated social status through clothing (Ratcliff et al., 2011) or biographical information (Dalmaso et al., 2012, 2014), finding that perceptual or knowledge-based cues can influence the priority of attention.

Although both perceptual and knowledge-based status cues have been shown to influence attention, it remains unclear whether they differ in the extent of their effects. One possibility is suggested by the levels-of-processing theory, which proposes that deeper, semantic processing leads to stronger cognitive effects (F. I. Craik & Lockhart, 1972; F. I. M. Craik & Tulving, 1975). From this perspective, knowledge-based status cues may exert a stronger influence on attention than perceptual cues, as they involve more elaborate semantic processing. An alternative account is provided by the dynamic interactive (DI) model of person perception, which proposes that social perception emerges from the continuous interplay between bottom-up perceptual inputs and top-down conceptual knowledge (Freeman & Ambady, 2011; Freeman & Johnson, 2016; Freeman et al., 2020). Within this framework, multiple sources of information—such as visual appearance and stored social knowledge—are simultaneously activated and mutually constrain one another, ultimately converging onto a unified representation of the target individual. From this perspective, perceptual cues and knowledge-based cues may be integrated within a common social representational space. As a result, despite their distinct origins, these different sources of status information may exert similar influences on downstream cognitive processes, including attentional allocation.

Despite these findings, several limitations remain in the existing literature. First, perceptual and knowledge-based status cues have typically been studied using different experimental paradigms, such as the Concentration-style game (Ratcliff et al., 2011) and gaze-cueing tasks (Dalmaso et al., 2012, 2014). This inconsistency makes it difficult to directly compare the magnitude of their effects on attention. In addition, these paradigms do not clearly separate the face as an object from its spatial location, making it unclear whether attentional effects are driven by object- or space-based mechanisms. To address these issues, the present study adopts the two-rectangle paradigm (Egly et al., 1994), which allows for a clear dissociation between space- and object-based attention. In this paradigm, participants view two parallel rectangles (either horizontal or vertical), with one end serving as the cued location. Targets then appear at the cued location (valid), the opposite end of the cued rectangle (invalid same-object, ISO), or an equidistant location on the uncued rectangle (invalid different-object, IDO). This paradigm produces two key effects: (1) faster responses in valid trials compared to ISO trials, reflecting a space-based attentional advantage where attentional efficiency is modulated by cue-target distance; and (2) faster responses in ISO trials compared to IDO trials, indicating an object-based attentional advantage whereby attending to one part of an object facilitates the processing of the entire object. Crucially, previous studies have elucidated the differing behavioral (Grignolio et al., 2024; Song et al., 2020; Zhao et al., 2020) and neural (Catak et al., 2022; He et al., 2004, 2008) mechanisms underlying space- and object-based attention. Space-based attention demonstrates relative stability, whereas object-based attention exhibits greater susceptibility to various factors, such as reward, facial expression and eye gaze (S. Hu et al., 2021; Song et al., 2021; Zhao et al., 2020). Furthermore, neurophysiological evidence suggests that object-based attention and social status processing may share overlapping neural mechanisms, particularly in regions such as the intraparietal sulcus (IPS) (Cavanagh et al., 2023; Cloutier et al., 2012; Zhang et al., 2017). This evidence further indicates that social status may modulate object-based attention.

Another critical but often overlooked issue concerns the temporal dynamics of how social status influences attentional allocation. Previous research suggests that the effect of social status on the gaze cueing effect emerges rapidly and decays quickly as the stimulus onset asynchrony (SOA) increases (Dalmaso et al., 2014), indicating that social status may primarily modulate early, relatively automatic stages of attentional processing. Notably, object-based attention itself is also sensitive to temporal factors. Prior studies have shown that the magnitude of object-based attentional effects varies as a function of SOA, typically increasing at shorter intervals and then decreasing at longer intervals, resulting in a non-linear, Gaussian-like pattern (Jeurissen et al., 2016). This suggests that object-based attention unfolds dynamically over time and may interact with socially relevant information at specific processing stages. Importantly, most existing studies examining the modulation of object-based attention by socially relevant factors—such as reward (Zhao et al., 2020), eye gaze (Song et al., 2020), and facial expressions (S. Hu et al., 2021)—have predominantly employed a single SOA (300 ms). While these studies demonstrate that high-level social information can influence object-based attention, they provide limited insight into when such modulation occurs and how long it persists. Moreover, it remains unclear whether perceptual and knowledge-based status cues follow similar or distinct temporal dynamics.

To address these gaps, the present study aimed to investigate whether and how perceptual and knowledge-based social status modulates selective attention. Specifically, we employed a modified two-rectangle paradigm (Egly et al., 1994; S. Hu et al., 2021; Song et al., 2021; Yan et al., 2022) with three SOA conditions (100, 300, and 500 ms). Faces served as stimuli, and their social status was manipulated via upper-body clothing (Experiment 1: perceptual status cues) or fictive biographies (Experiment 2: knowledge-based status cues). A supplementary experiment (Experiment S1) was conducted to verify that faces could reliably elicit both space- and object-based attention (see Supplemental Materials). Based on prior evidence, we tested whether social status derived from both types of cues would selectively modulate object-based attention, particularly at intermediate processing stages (e.g., the 300 ms SOA).

## 2. Materials and Methods

### 2.1. Participants

The sample size was determined using power analyses based on the effect sizes reported in previous studies. Specifically, we referenced the interactions between cue position and validity (S. Hu et al., 2021: *η*_p_^2^ = 0.26 and *η*_p_^2^ = 0.18; Song et al., 2021: *η*_p_^2^ = 0.16), as well as between validity and SOA (Song et al., 2020: *η*_p_^2^ = 0.19 and *η*_p_^2^ = 0.25). A power analysis conducted using MorePower (V6.0.4; Campbell & Thompson, 2012) indicated that a minimum sample size of 28 participants would be sufficient to detect the smallest effect (*η*_p_^2^ = 0.16) with 80% power at an alpha level of 0.05. To account for potentially noisier data, we increased the sample size to 30 participants per experiment.

In Experiment 1, 30 volunteers (13 males; mean age = 19.63 ± 1.76 years), who were naive to the experimental purpose, participated in the study. In Experiment 2, a separate group of 30 undergraduate students (12 males; mean age = 19.81 ± 1.49 years) was recruited. All participants provided written informed consent prior to their participation and reported normal or corrected-to-normal visual acuity.

### 2.2. Apparatus and Stimuli

All experiments were conducted in a sound-attenuated room. Stimuli were presented on a 19-inch color monitor with a refresh rate of 60 Hz, positioned 63 cm from the participants. Stimulus presentation and data collection were controlled using E-Prime 2.0.

In Experiment 1, the stimuli consisted of four male faces and four types of upper-body clothing. The faces, along with eight additional grayscale faces displaying neutral expressions and a direct gaze, were selected from the Chinese Facial Affective Picture System (CFAPS; Gong et al., 2011). Twenty independent volunteers rated these faces on valence, arousal, dominance, and attractiveness using 9-point scales (1 = not at all, 9 = extremely). One-sample and paired-samples *t* tests confirmed the suitability of the four experimental faces (see Tables S1 and S2). Social status was manipulated via clothing, with four types of clothing representing high or low status selected from online sources. Using Photoshop, grayscale composite images were created by pairing two faces with high-status clothing and two with low-status clothing. The face-clothing pairings were counterbalanced across participants.

In Experiment 2, only facial stimuli were presented, and social status was manipulated using fictive biographical information. Two sets of status descriptions were used: in half of the trials, participants viewed “Dean of a University” (high status) and “Unemployed Worker from the Countryside” (low status), whereas in the remaining trials, they viewed “Official of a Subsidiary” (high status) and “Water Delivery Worker” (low status). All face stimuli were presented upright to preserve ecological validity and maintain typical face-processing characteristics. All experimental stimuli are available in the OSF repository (see Supplemental Materials).

In both experiments, the trials of the probe detection task began with a central black fixation (0.4° × 0.4°) presented on a gray background (RGB: 211, 211, 211), flanked by two stimuli positioned horizontally to the left and right of the fixation. Each stimulus subtended 2.4° × 5.8° of visual angle, with a separation of 1° between them. A yellow outline served as the cue, and a red dot (0.5° × 0.5°) was used as the probe.

### 2.3. Procedure and Design

In both Experiment 1 and Experiment 2, participants completed a practice session followed by an experimental session. The practice session consisted of a subset of trials from the probe detection task, and participants were required to achieve at least 90% accuracy before continuing. The experimental session included the probe detection task followed by a status-checking phase.

**Figure 1:**
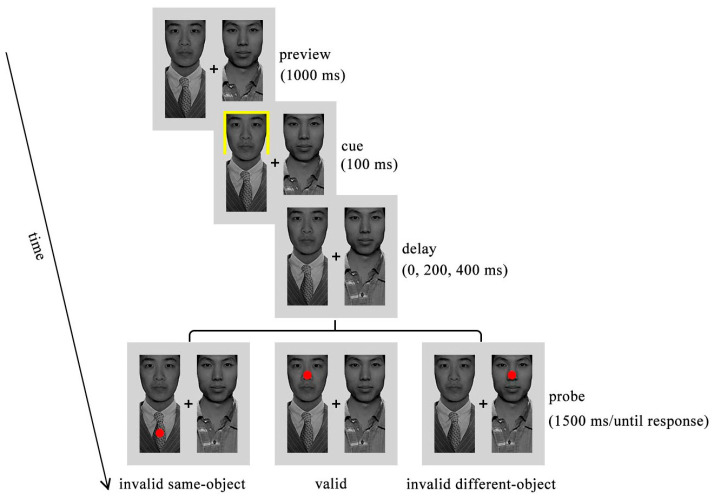
Schematic illustration of the probe detection task in Experiments 1 and 2. Notably, Experiment 2 employed faces only as stimuli. The target appeared at the cued location in the valid trials, or at the uncued end of the cued face in the invalid same-object (ISO) trials, or at the uncued faces in the invalid different-object (IDO) trials.

In the probe detection task, participants were required to maintain fixation on a central cross and respond to the probe as quickly and accurately as possible (see Figure 1). Each trial began with the presentation of high- and low-status faces aligned with the fixation for 1000 ms, followed by a cue presented randomly at one of the four ends of the two faces for 100 ms. After a delay of 0, 200, or 400 ms, a red dot (or no target in catch trials) appeared and remained on the screen for up to 1500 ms or until a response was made by pressing the “M” key. The next trial began after a 1000 ms inter-trial interval. The positions of high- and low-status faces were counterbalanced across blocks. Reaction times (RTs) and accuracy were recorded. Trials were classified as incorrect if participants missed a target probe or responded during catch trials in which no probe was presented.

Following the probe detection task, participants completed a status checking phase to assess the effectiveness of the status manipulation. Participants rated the perceived status of each face using a 9-point scales (1 = extremely low status, 9 = extremely high status) by pressing the corresponding number keys.

Both experiments employed a similar procedure and design, with Experiment 2 including an additional learning phase prior to the probe detection task.

In Experiment 2, participants first completed a learning phase in which two faces were presented on a black screen, each paired with a fictive biography. One face was associated with relatively high status, whereas the other was associated with low status. The biographical information was counterbalanced across participants. This phase was self-paced. Following the learning phase, participants completed a 12-item learning test in which they judged whether the presented biographical information correctly matched the previously learned faces (“1” = “yes”, “2” = “no”). Novel faces selected from the CFAPS (Gong et al., 2011) were included as distractors. Participants were required to achieve at least 90% accuracy before proceeding to the next phase.

The probe detection task followed a 2 (cue position: high status, low status) × 3 (cue validity: valid, ISO, IDO) × 3 (SOA: 100, 300, 500 ms) within-subjects factorial design. Each participant completed 20 practice trials and 960 target-present trials, along with 192 catch trials. Among the target-present trials, 576 trials (60%) were valid, and 192 trials (20%) were included in each invalid condition.

## 3. Results

### 3.1. Status Manipulation Check

Statistical analyses were conducted using JASP (V0.19.3.0) and SPSS (V26.0). The mean social status ratings for Experiment 1 and Experiment 2 are reported in Table 1 and Table 2, respectively. Both one-sample and paired-samples *t* tests confirmed significant differences between the high- and low-status conditions, validating the effectiveness of the status manipulation.

### 3.2. Analysis of RTs

For Experiment 1, incorrect responses (1.12%) and RTs exceeding three standard deviations from the mean (2.08%) were excluded from the analyses. For Experiment 2, incorrect responses (0.72%) and RTs exceeding three standard deviations from the mean (2.01%) were also excluded from the analyses.

#### 3.2.1. Experiment 1

To examine the impact of social status on space-based attention, a 2 (cue position: high status, low status) × 2 (validity: valid, ISO) × 3 (SOA: 100, 300, 500 ms) repeated-measures analysis of variance (ANOVA) was conducted on mean RTs. The results showed a significant main effect of validity, *F*(1, 29) = 6.95, *p* = 0.013, *η*_p_^2^ = 0.19, whereby RTs were faster for the valid condition (*M* = 324 ms, *SE* = 6.73) compared with the ISO condition (*M* = 327 ms, *SE* = 7.18). The main effect of cue position was significant, *F*(1, 29) = 15.18, *p* < 0.001, *η*_p_^2^ = 0.34, indicating that RTs were faster for high status (*M* = 325, *SE* = 6.99) compared with low status (*M* = 327, *SE* = 6.89). The main effect of SOA was significant, *F*(2, 58) = 225.71, *p* < 0.001, *η*_p_^2^ = 0.89. Post hoc test showed that RTs were faster for the 300 ms SOA condition (*M* = 302 ms, *SE* = 6.39) compared with both the 100 (*M* = 351 ms, *SE* = 7.42) and 500 ms (*M* = 324 ms, *SE* = 7.34) SOA conditions, *t*s > 9.80, *p*s < 0.001; additionally, RTs for 500 ms SOA condition were faster than those for 100 ms SOA condition, *t*(1, 29) = 11.43, *p* < 0.001, Cohen’s *d* = 0.67. No other significant effects were found, *F*s < 0.81, *p*s > 0.374. These results suggest that social status at the perceptual level had no impact on space-based attention.

To examine the impact of social status on object-based attention, a three-way ANOVA was conducted on RTs as the dependent variable, with cue position (2: high status, low status), validity (2: ISO, IDO), and SOA (3: 100, 300, 500 ms) as within-subject factors. The results showed a significant main effect of validity, *F*(1, 29) = 9.54, *p* = 0.004, *η*_p_^2^ = 0.25, whereby RTs were faster for the ISO condition (*M* = 327 ms, *SE* = 7.18) compared with the IDO condition (*M* = 330 ms, *SE* = 7.54). The main effect of SOA was significant, *F*(2, 58) = 168.67, *p* < 0.001, *η*_p_^2^ = 0.85. Post hoc test showed that RTs were faster for 300 ms SOA condition (*M* = 307 ms, *SE* = 6.91) compared with both the 100 (*M* = 353 ms, *SE* = 7.78) and 500 ms (*M* = 325 ms, *SE* = 7.74) SOA conditions, *t*s > 7.30, *p*s < 0.001; additionally, RTs for 500 ms SOA condition were faster than those for 100 ms SOA condition, *t*(1, 29) = 10.95, *p* < 0.001, Cohen’s *d* = 0.67. The two-way interaction between validity and SOA was significant, *F*(2, 58) = 3.57, *p* = 0.035, *η*_p_^2^ = 0.11. Simple main effects analyses showed that for the 300 ms SOA condition, the difference in RTs between the ISO (*M* = 303 ms, *SE* = 6.59) and IDO (*M* = 310 ms, *SE* = 7.32) conditions was significant, *F*(1, 29) = 15.13, *p* < 0.001; in contrast, no similar result was found for both the 100 and 500 ms SOA conditions, *F*s < 1.18, *p*s > 0.287. No other significant main effects or two-way interactions were found, *F*s < 1.84, *p*s > 0.169.

**Figure 2:**
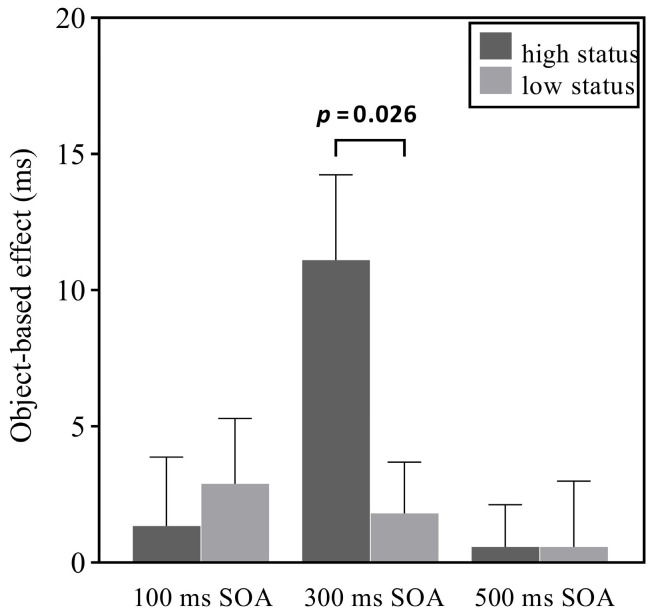
The results of Experiment 1. Object-based effect (OBE) as a function of cue position and SOA. Error bars denote *SE*s of the mean.

Importantly, the three-way interaction between cue position, validity, and SOA was significant, *F*(2, 58) = 3.77, *p* = 0.029, *η*_p_^2^ = 0.12. Therefore, we conducted a 2 (cue position: high status, low status) × 3 (SOA: 100, 300, 500 ms) repeated-measures ANOVA with the object-based effect (OBE) as the dependent variable (calculated by subtracting RTs in ISO trials from IDO trials). The results showed a significant main effect of SOA, *F*(2, 58) = 3.57, *p* = 0.035, *η*_p_^2^ = 0.11. Post hoc tests showed that the OBE for the 500 ms SOA condition (*M* = 1 ms, *SE* = 1.25) was smaller than that for the 300 ms SOA condition (*M* = 6 ms, *SE* = 1.66), *t*(1, 29) = 2.66, *p* = 0.037, Cohen’s *d* = 0.45; in contrast, the OBE did not differ significantly between the 100 (*M* = 2 ms, *SE* = 1.96) and 500 ms SOA conditions, *t*s < 1.99, *p*s > 0.113. The main effect of cue position was not significant, *F*(1, 29) = 1.22, *p* = 0.278, *η*_p_^2^ = 0.04. The interaction between cue position and SOA was significant (see Figure 2), *F*(2, 58) = 5.55, *p* = 0.026, *η*_p_^2^ = 0.16. Simple main effects analyses showed that for the 300 ms SOA, the difference in the OBE between the high- and low-status conditions was significant, *F*(1, 29) = 5.55, *p* = 0.026; however, no similar results were found for 100 and 500 ms SOA conditions, *F*s < 0.27, *p*s > 0.609. These findings indicate that social status had an impact on object-based attention at the 300 ms SOA.

Accuracy analyses revealed no significant main effects or interactions, *F*s < 1, *p*s > 0.05, indicating no speed–accuracy trade-off.

#### 3.2.2. Experiment 2

To examine the impact of social status on space-based attention, a three-way repeated-measures ANOVA was conducted on RTs as the dependent variable, with cue position (2: high status, low status), validity (2: valid, ISO), and SOA (3: 100, 300, 500 ms) as within-subject factors. The results showed a significant main effect of validity, *F*(1, 29) = 13.19, *p* < 0.001, *η*_p_^2^ = 0.31, whereby RTs were faster for the valid condition (*M* = 319 ms, *SE* = 5.15) compared with the ISO condition (*M* = 324 ms, *SE* = 4.92). The main effect of cue position was significant, *F*(1, 29) = 9.09, *p* = 0.005, *η*_p_^2^ = 0.24, indicating that RTs were faster for high status (*M* = 320, *SE* = 5.01) compared with low status (*M* = 323, *SE* = 5.01). The main effect of SOA was significant, *F*(2, 58) = 105.37, *p* < 0.001, *η*_p_^2^ = 0.78. Post hoc test showed that RTs were faster for the 300 ms SOA condition (*M* = 300 ms, *SE* = 4.73) compared with both the 100 (*M* = 344 ms, *SE* = 6.19) and 500 ms (*M* = 319 ms, *SE* = 4.83) SOA conditions, *t*s > 8.72, *p*s < 0.001; additionally, RTs for 500 ms SOA condition were faster than those for the 100 ms SOA condition, *t*(1, 29) = 6.34, *p* < 0.001, Cohen’s *d* = 0.83. No other significant two-way interactions were found, *F*s < 3.00, *p*s > 0.058. Importantly, the three-way interaction between cue position, validity, and SOA was not significant, *F*(2, 58) = 2.29, *p* = 0.111.

To examine the impact of social status on object-based attention, a three-way ANOVA was conducted on RTs as the dependent variable, with cue position (2: high status, low status), validity (2: ISO, IDO), and SOA (3: 100, 300, 500 ms) as within-subject factors. The results showed a significant main effect of validity, *F*(1, 29) = 34.65, *p* < 0.001, *η*_p_^2^ = 0.54, whereby RTs were faster for the ISO condition (*M* = 324 ms, *SE* = 4.92) compared with the IDO condition (*M* = 331 ms, *SE* = 5.09). The main effect of SOA was significant, *F*(2, 58) = 103.54, *p* < 0.001, *η*_p_^2^ = 0.78. Post hoc tests showed that RTs were faster for the 300 ms SOA condition (*M* = 307 ms, *SE* = 4.97) compared with both the 100 (*M* = 351 ms, *SE* = 6.09) and 500 ms (*M* = 324 ms, *SE* = 4.63) SOA conditions, *t*s > 7.34, *p*s < 0.001; additionally, RTs were faster for the 500 ms SOA condition compared with the 100 ms SOA condition, *t*(1, 29) = 6.69, *p* < 0.001, Cohen’s *d* = 0.89. The two-way interaction between cue position and SOA was significant, *F*(2, 58) = 8.94, *p* < 0.001, *η*_p_^2^ = 0.24. No other significant main effects or two-way interactions were found, *F*s < 2.93, *p*s > 0.098.

**Figure 3:**
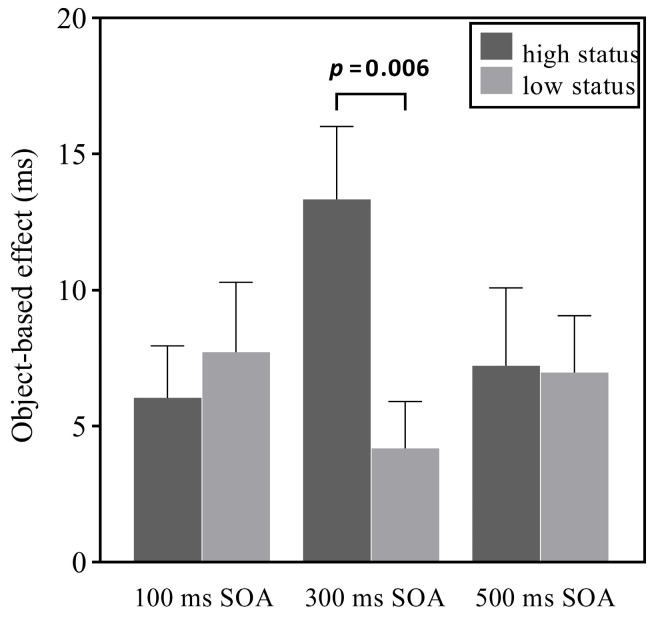
The results of Experiment 2. Object-based effect (OBE) as a function of cue position and SOA. Error bars denote *SE*s of the mean.

Importantly, the three-way interaction between cue position, validity, and SOA was significant, *F*(2, 58) = 5.40, *p* = 0.007, *η*_p_^2^ = 0.16. Therefore, we conducted a 2 (cue position: high status, low status) × 3 (SOA: 100, 300, 500 ms) repeated-measures ANOVA with the OBE as the dependent variable. The results showed a significant interaction between cue position and SOA (see Figure 3), *F*(2, 58) = 5.40, *p* = 0.007, *η*_p_^2^ = 0.16. Simple main effects analyses showed that only for the 300 ms SOA, the difference in the OBE between high- and low-status conditions was significant, *F*(1, 58) = 8.82, *p* = 0.006; in contrast, no similar results were found for the100 and 500 ms SOA conditions, *F*s < 0.54, *p*s > 0.468. These findings indicate that social status had an impact on object-based attention at 300 ms SOA. No other significant main effects were found, *F*s < 2.08, *p*s > 0.160.

Accuracy analyses revealed no significant main effects or interactions, *F*s < 1, *p*s > 0.05, indicating no speed–accuracy trade-off.

#### 3.2.3. Cross-Experimental Analysis

To examine the extent to which perceptual and knowledge-based social status influence object-based attention, a 2 × 2 × 3 repeated-measures ANOVA was conducted on the OBE, with cue position (2: high status, low status) and SOA (3: 100, 300, 500 ms) as within-subject factors and experiment (2: Experiment 1, Experiment 2) as a between-subject factor. The results showed the three-way interaction between cue position, SOA and experiment was not significant, *F*(2, 116) = 0.004, *p* = 0.996. A Bayesian repeated-measures ANOVA further showed strong evidence against the three-way interaction between cue position, SOA, and experiment (BF_incl_ = 0.037). A 2 × 2 repeated-measures ANOVA for the 300 ms SOA also showed that the interaction between cue position and experiment was not significant, *F*(1, 58) = 0.001, *p* = 0.975. The corresponding Bayesian analysis yielded a BF_incl_ of 0.749. Together, these results indicate that perceptual and knowledge-based status cues have a similar effect on object-based attention at the 300 ms SOA.

## 4. Discussion

This study investigated how different types of status cues (perceptual cues vs. knowledge-based cues) influence early-stage selective attention processing. The results showed that participants exhibited enhanced attentional vigilance toward high-status individuals. Crucially, we found converging evidence that both perceptual (Experiment 1) and knowledge-based (Experiment 2) status cues modulated object-based attention to a comparable extent at the 300 ms SOA. In contrast, space-based attention remained unaffected by social status. These findings provide direct evidence for a dissociation between space- and object-based attention, suggesting that social status selectively modulates object-based attentional processing.

Across two experiments, we consistently found that both perceptual and knowledge-based social status cues modulated object-based attention. At the perceptual level, clothing, as a costly signal, externalizes an individual’s resource-holding potential, thereby providing rapid and intuitive cues to social status (Mattan & Kubota, 2020; Oh et al., 2020; Ratcliff et al., 2011). At the knowledge-based level, keywords in biographical information (e.g., “Dean of a University”) can rapidly activate high-status schemas (Dalmaso et al., 2012, 2014; Mattan et al., 2017; Schwartz & Yovel, 2016). The observed attentional patterns likely reflect adaptive social evaluation criteria for identifying potential interaction partners. Such heightened attention may stem from high-status individuals’ disproportionate control over material and social resources (Breton et al., 2014; Magee & Galinsky, 2008), or from the potentially greater consequences of engaging with individuals in positions of power (Ames & Fiske, 2013; Haaker et al., 2016). These findings are consistent with prior research showing that both perceptual cues (e.g., eye contact: Song et al., 2021) and semantic information (e.g., Chinese words: S. S. Hu et al., 2020, 2023; Li et al., 2025) can modulate object-based attention. This convergence is further supported by evidence of overlapping neural representations underlying object-based attention and social status (Cavanagh et al., 2023; Cloutier et al., 2012; Mattan et al., 2017; Zhang et al., 2017).

Importantly, the present findings showed that perceptual and knowledge-based status cues exerted highly similar effects on object-based attention. This pattern is consistent with the DI account of person perception, which posits that bottom-up perceptual inputs and top-down conceptual knowledge are continuously integrated through reciprocal interactions (Freeman & Ambady, 2011; Freeman & Johnson, 2016; Freeman et al., 2020). Although these two types of status cues were manipulated in separate experimental contexts, their convergent effects suggest that perceptual and knowledge-based status cues may give rise to comparable representational states. Within the DI framework, appearance-based (e.g., clothing) information and knowledge-based (e.g., fictive biographies) information can be incorporated into a unified representation of social status via mutual constraint between perceptual and conceptual processes. Once formed, such integrated representations may guide attentional selection in a similar manner, regardless of the specific form in which the status information is conveyed. More broadly, these findings suggest that attentional allocation in social contexts may be driven less by the specific type of status cue, and more by the abstract representation that emerges from the integration of multiple information sources. This interpretation is further compatible with the common currency framework, which proposes that diverse types of information can be transformed into a shared, abstract representational scale that supports selection processes (Levy & Glimcher, 2012; Sescousse et al., 2015; Zink et al., 2008). Consequently, the present study provides empirical support for the DI account by demonstrating that distinct inputs can lead to functionally equivalent effects on attentional selection.

Notably, the object-based attentional advantage emerged only at the 300 ms SOA, indicating that the influence of social status is rapid but transient. The significant effect observed at the 300 ms SOA likely reflects the temporal characteristics of object-based attentional processing. Previous research has demonstrated that object-based attentional effects vary nonlinearly across SOAs, typically following a Gaussian-like distribution, with effects increasing at intermediate intervals and diminishing at longer delays (Jeurissen et al., 2016). In the present study, the inclusion of three SOA conditions (100, 300, and 500 ms) allowed us to capture this temporal profile, with the strongest effect observed at 300 ms—corresponding to the peak of this distribution. This pattern is consistent with prior findings showing that socially relevant cues, such as eye contact, also exert a maximal influence on object-based attention at intermediate SOAs (Yan et al., 2022). Moreover, the transient nature of this effect aligns with previous research on social attention, which suggests that the attentional advantage of high-status individuals emerges rapidly and decays quickly over time (Dalmaso et al., 2012, 2014). Together, these findings suggest that social status modulates object-based attention within a temporally constrained window, primarily during the early stages of attentional selection.

Building on these findings, we propose the Global Workspace Integration Hypothesis of Social Status Processing (GWIH-SSP) as a unifying account of how social status may shape attentional selection. Within this framework, the brain’s global workspace may transform diverse types of status information into an abstract representation of status salience that becomes available to downstream cognitive systems. Critically, the temporally specific emergence of status effects may operate at an intermediate stage where integrated representations of social salience can effectively modulate object-based attention rather than space-based attention. High status, whether conveyed perceptually or through knowledge-based information, may be represented as highly salient within the global workspace, potentially gaining prioritized access to the attentional system. Such a mechanism could support efficient social behavior by enabling rapid and flexible responses to high-status individuals in varying informational contexts. Nevertheless, the present framework should be considered a theoretical interpretation of the current findings rather than a directly tested model. Future studies combining behavioral and neurophysiological measures (e.g., EEG and fMRI) with computational modeling approaches will be needed to further evaluate the validity of this framework and clarify the mechanisms underlying status-based attention.

Several limitations should be noted. First, perceptual and knowledge-based status cues were examined in separate experiments. Although this design allowed us to isolate the effect of each type of status information, it remains unclear how these cues interact when they are simultaneously available. Future studies could use factorial designs to examine whether perceptual and conceptual status cues exert additive or interactive effects on attentional selection. Second, the present study used a relatively homogeneous sample of undergraduate students and included only male face stimuli. Future research should test more diverse participant groups and stimulus identities to examine the generalizability of the present findings as well as possible interactions between gender and social status. Finally, the current findings were obtained using laboratory-based RT tasks, which may not fully capture attentional processes in real social contexts. Future studies could combine more naturalistic paradigms with methods such as eye tracking or electrophysiological measures to further investigate the mechanisms underlying status-based attentional selection.

## 5. Conclusions

In conclusion, the present study demonstrates that both perceptual and knowledge-based status cues exert comparable effects on object-based attention at an intermediate processing stage (300 ms SOA), while leaving space-based attention unaffected. These findings suggest that social status operates as a higher-order modulatory factor that selectively influences object-based attentional selection. Although this attentional advantage for high-status individuals is transient, it reflects a rapid and adaptive mechanism for prioritizing socially relevant targets. Overall, this study advances our understanding of how social status shapes early attentional processes and highlights the role of abstract social value representations in guiding human attention.

## Figures and Tables

**Table 1:** Mean social status ratings (and standard deviations, *SD*) for Experiment 1.

	Status	*M* ± *SD*	One-Sample *t*	Paired-Samples *t*
pair 1	high status	6.63 ± 0.96	9.28 ***	8.86 ***
	low status	4.17 ± 0.87	5.22 ***	
pair 2	high status	6.80 ± 1.00	9.89 ***	9.16 ***
	low status	4.03 ± 1.25	4.25 ***	

Note. *** *p* < 0.001.

**Table 2:** Mean social status ratings (and standard deviations, *SD*) for Experiment 2.

	Status	*M* ± *SD*	One-Sample *t*	Paired-Samples *t*
pair 1	high status	7.33 ± 1.16	11.07 ***	16.35 ***
	low status	3.23 ± 0.82	11.84 ***	
pair 2	high status	7.33 ± 1.21	10.54 ***	18.68 ***
	low status	3.57 ± 0.73	10.79 ***	

Note. *** *p* < 0.001.

## Data Availability

The data are available from the corresponding authors on reasonable request.

## Supplementary Materials

The following supporting information can be downloaded at: https://osf.io/bnyju/overview?view_only=c5417af5849844d7b1c00ba14fa38004 (accessed on 13 May 2026), Supplementary Materials.docx: methods and results of Experiment S1; Exps_Stimuli folder: experimental stimuli.
